# Demographic and microbial characteristics of extrapulmonary tuberculosis cases diagnosed in Malatya, Turkey, 2001-2007

**DOI:** 10.1186/1471-2458-11-154

**Published:** 2011-03-08

**Authors:** Selami Gunal, Zhenhua Yang, Mansi Agarwal, Mehmet Koroglu, Zeynep Kazgan Arıcı, Riza Durmaz

**Affiliations:** 1 Department of Clinical Microbiology, Medical Faculty, Inonu University, Malatya, Turkey; 2 Department of Epidemiology, University of Michigan, Ann Arbor, Michigan 48109, USA; 3 Malatya Governor Research Hospital, Microbiology Laboratory, Malatya, Turkey; 4 Tuberculosis Control Dispensary, Malatya, Turkey

## Abstract

**Background:**

Extrapulmonary tuberculosis (EPTB) has an increasing rate in Turkey. The reason remains largely unknown. A better understanding of the demographic and microbial characteristics of EPTB in the Turkish population would extend the knowledgebase of EPTB and allow us to develop better strategies to control tuberculosis (TB).

****Methods**:**

We retrospectively evaluated clinical and laboratory data of 397 bacteriologically-confirmed TB cases diagnosed during an eight year-period using by chi-square analysis and multivariate logistic regression model.

**Results:**

Of the 397 study patients, 103 (25.9%) had EPTB and 294 (74.1%) had pulmonary tuberculosis (PTB). The most commonly seen two types of EPTB were genitourinary TB (27.2%) and meningeal TB (19.4%). TB in bone/joints, pleural cavity, lymph nodes, skin, and peritoneal cavity occurred at a frequency ranging from 9.7% to 10.7%. The age distribution was significantly different (P < 0.01) between PTB and EPTB, with patients older than 45 years tending to have an increased risk of EPTB. Furthermore, the distribution of different types of EPTB differed significantly among age groups (P = 0.03). Meningeal and bone and/or joint TB were more commonly observed among the male patients, while lymphatic, genitourinary, and peritoneal TB cases were more frequently seen among females. Unique strain infection was statistically significantly associated with EPTB (OR: 2.82, 95% CI [1.59, 5.00])

**Conclusions:**

EPTB accounted for a significant proportion of TB cases in Malatya, Turkey between 2001 and 2007. The current study has provided an insight into the dynamics of EPTB in Malatya, Turkey. However, the risk factors for having EPTB in Malatya, Turkey remain to be assessed in future studies using population-based or randomly selected sample.

## Background

Tuberculosis (TB) remains one of the leading infectious diseases causing significant morbidity and mortality worldwide. One third of the world's population is latently infected with *Mycobacterium tuberculosis *[1], of which about 10% may develop active disease at anytime [2]. Although the infection of *M. tuberculosis *usually results in pulmonary TB, other organs and tissues can also be affected, resulting in extrapulmonary or disseminated TB [3-5].

Extrapulmonary TB (EPTB) is a significant health problem, as is pulmonary TB (PTB), in both developing and developed countries [4,6]. For example, in India, while 15-20 percent of the immunocompetent adult TB cases were EPTB, the rate of EPTB was increased to more than 50% among the HIV co-infected patients [7]. In the Netherlands, the frequency of EPTB was found to be 15% among the eastern and central Europeans, 58.9% among the Somali, 36.6% among people of other African origins, and 44% among the Asians [8]. The reported proportions of EPTB among all TB cases in other developed countries ranged from 12% to 28.5% [4,9-12].

Turkey is a developing country with a population of more than 70 million and about 20,000 notified new TB cases annually. The proportion of EPTB among all TB cases in Turkey had increased from 19.6% in 1996 to 32.5% in 2007 [13]. However, the reason for such an increase remains largely unknown. A directly observed therapy short-course strategy was started as a pilot study in 2003 and it was implemented throughout the country in 2008. BCG vaccination has been routinely applied to all children. TB control is mainly done by dispensaries around the country, sanatorium hospitals, and university hospitals. In TB dispensaries, the diagnosis of TB is usually based on a combination of medical history, physical examination, chest-X ray, and microscopic examination of clinical specimens for the presence of tubercle bacilli. People suspected to have TB will then be refered to the university or sanatorium hospitals, where mycobacterial culture can be performed for confirmation of the diagnosis. A limited number of medical centers are able to perform accurate and rapid culture and susceptibility testing. All TB patients are treated free-off charge.

Malatya is the third biggest city in the eastern Anatolia region of Turkey. The TB incidence rate (28.5/100.000) in Malatya has been higher than the average of the country and the EPTB rate which is higher than the average of the country has increased steadily in recent years, from 33.3% in 2005 to 42.2% in 2007 [13]. The present study was conducted to gain insight into the demographic and microbial characteristics of EPTB cases in Malatya, Turkey, thereby to extend the knowledgebase of EPTB based on which better TB control strategies can be developed.

## Methods

### Patient population

The study sample included 397 TB patients whose TB diagnosis were confirmed by mycobacterial culture performed at Turgut Ozal Medical Center, Inonu University, Malatya, Turkey, during the time period from January, 1^st ^2001 to December 31^st^, 2007. Turgut Ozal Medical Center is one of the five medical institutions located in the city of Malatya and it provides medical serives to the 850,000 population residing in Malatya, Turkey. The other four of the five medical institutions include two TB dispensaries and two governmental hospitals. The initial diagnosis of TB was made in any of the five medical institutions. An individual was suspected to have active pulmonary TB (PTB) if the individual had pulmonary TB symptoms (productive, prolonged cough of three or more weeks, chest pain, the production of sputum, and hemoptysis) and systemic symptoms (low grade remittent fever, chills, night sweats, appetite loss, weight loss, easy fatiguability). Information about prior TB exposure, infection or disease, past TB treatment was obtained as additional supporting evidence. A PPD tuberlin skin test and chest X-ray were done for TB suspects at local TB dispensaries. The initial diagnosis of EPTB cases was generally considered when a patient complained of organ-specific symptoms, besides having the systemic symptoms (described above) and sometimes concurrently present pulmonary TB symptoms, and the medical history supporting TB diagnosis described above. For confirmation of TB diagnosis, appropriate clinical specimens for each form of TB, such as sputum, cerebrospinal fluid, urine, biopsy materials were examined by microscopy, mycobacterial culture, TB-PCR test and sometimes histopathologic testing. Mycobacterial culture was only done at Turgut Ozal Medical Center, the university hospital. Each year, 200-270 new TB cases are notified in the entire Maltya province, of which 80-90 were mycobaterial culture-positive. The study sample included all the culture-positive cases confirmed at Turgut Ozal Medical Center during the study period, representing approximately 58% of the culture-positive TB cases in Malatya during the study period. The demographic (age and sex), clinical (type of TB diagnosis), and laboratory data (pathologic, microscopic, culture and molecular analysis of *M. tuberculosis *isolates) of the patients were retrieved from the existing molecular epidemiological study database established previously by Durmaz et al [14].

No patient identifier was included in the database used for the study. The study protocol was approved by the University of Michigan's Institutional Review Board and the Inonu University Ethical Committee.

### *M. tuberculosis *isolates

One *M. tuberculosis *isolate from each patient was included in this study. The identification of *M. tuberculosis *isolates was done by conventional methods, including the nitrate reduction test, the niacin accumulation test, the BACTEC *p*-nitro-α-acetylamino-β-hydroxypropiophenone test (Becton Dickinson), and growth characteristics [15]. Susceptibility testing to isoniazid (0.1 μg/ml), rifampin (2 μg/ml), streptomycin (2 μg/ml), and ethambutol (2.5 μg/ml) was performed by the modified 1% proportion method in the BACTEC 460 radiometric system or the MGIT 9600 system (Becton Dickinson, Sparks, Maryland, USA). The isolates that are resistant to at least isoniazid and rifampin were defined as multidrug resistant (MDR) [16].

### Genotyping of the study isolates

The IS*6110 *restriction-fragment-length-polymorphism (RFLP) was performed using standard methods [17]. The banding patterns of the isolates were compared by using Bionumerics (Applied Maths, Sint-Martens-Latem, Belgium). Standard spoligotyping was performed with the Dra and Drb primers [18]. Spoligotype of each strain was compared to the international spoligotyping database of the Pasteur Institute of Guadeloupe, and major phylogenetic clades were discribed according to signatures provided in SpolDB4 [19]. Clusters were determined if any two or more isolates having identical IS*6110 *fingerprinting patterns comprising more than 5 bands (high copy) or having identical IS*6110 *fingerprinting patterns comprising 5 or less bands (low-copy) and identical spoligotyping patterns.

### Statistical analyses

We compared the distributions of demographic (age and sex) and microbial characteristics (drug susceptibility, IS*6110 *copy number range, genotyping-based clustering, and the major spoligotypes) between PTB and EPTB groups by chi-square analysis. We defined EPTB versus PTB, based on the affected anatomic sites from which we isolated M. tuberculosis. EPTB group included cases whose isolates were obtained from any organs and tissues, except for lung parenchyma. Intrathoracic lymphatic TB without a concurrent diagnosis of PTB was defined as EPTB [12]. The PTB group contained the cases having positive sputum cultures. P value ≤ 0.05 was regarded as statistically significant. Multivariate logistic regression was performed to identify independent risk factors for having EPTB. All of the statistical analyses were done using SAS, version 9.0 (SAS Institute, Cary, NC).

## Results

### Characteristics of the study patients

Of the 397 patients, 238 (59.9%) were males, 159 (40.1%) were females. The male to female ratio was 1.5. The majority of the patients (83%) were born in Malatya, the remaining 17% were from neighboring cities of Malatya. The median age of the study patient was 32 years (ranging from less than 1 year to 89 years).

### Frequency distribution of different types of EPTB

One hundred and three (25.9%) were defined as having EPTB and the remaining 294 (74.1%) as having pulmonary TB, based on the sites from which the isolates were obtained. Of the 103 EPTB, the most commonly seen type was genitourinary TB (28 cases; 27.2%), followed by meningeal TB (20 cases; 19.4%), bone and/or joint TB (11 cases; 10.7%), pleural TB (11 cases; 10.7%), lymphatic TB (10 cases; 9.7%), skin TB (10 cases; 9.7%), peritoneal TB (10 cases; 9.7%), pericardial TB (2 cases; 1.9%), and miliary TB (1 case; 0.9%).

### Demographic characteristics of EPTB cases

**Figure 1:**
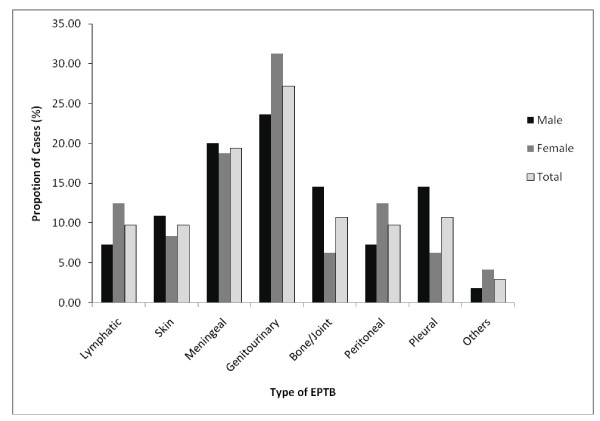
**Overal and gender-specific proportional distributions of the different types of EPTB**. (P = 0.54).

**Figure 2:**
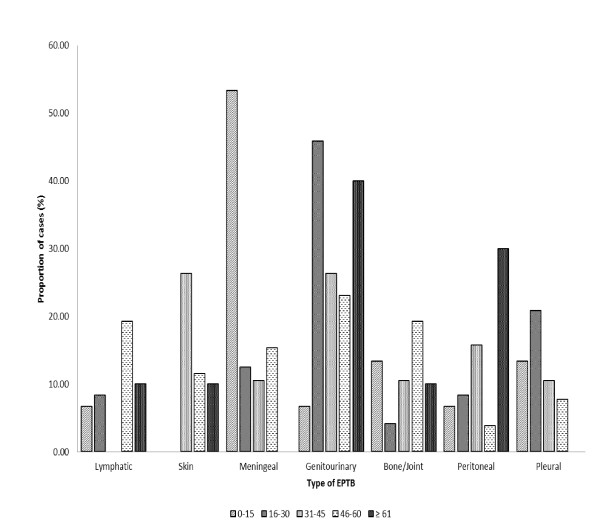
**Age-specific proportional distributions of different types of EPTB by age groups (P = 0.03)**.

Among the 103 EPTB cases, women and men each accounted for half of the cases (Table 1). The male to female ratio for PTB (1.64) was higher than that for EPTB (1.14). The median age for PTB was 30 years (ranging from 2 to 89), while the median age for EPTB was 33 years (ranging from < 1 to 80). Although the overall distributions of the different types of EPTB was not statistically significant between females and males, pleural, meningeal and bone/joint TB appeared to be more commonly observed among the male patients while lymphatic, genitourinary, and peritoneal TB cases were predominant among females (Figure 1). Furthermore, the distributions of the different types of EPTB differed significantly among different age groups (Figure 2). In the youngest age group (< 1-15 years), meningeal TB was the most frequently seen type of EPTB, accounting for more than half of the EPTB cases in that age group. In contrast, in the 16-30 year-age group, the most commonly seen type was genitourinary, whereas for the remaining three age groups (31-45, 46-45, and ≥ 61 years), the most commonly seen types were skin, lymphatic and bone/joint, and peritoneal, respectively (Figure 2). Because of the age-specific distribution of the specific types of EPTB, the age composition differed among different types of EPTB (P = 0.03). For example, while lymphatic and bone/joint TB were predominated by people in the age group 46 - 60 year old, meningeal TB contained mainly patients younger than 15 years, peritoneal TB was predominated by patients in the 31-45 years and ≥ 61 year age groups; genitourinary and pleural in the 16-30 years age group, skin in the 31-45 years age group.

**Table 1:** Distribution of demographic and microbial characteristics among 294 pulmonary and 103 extrapulmonary TB cases diagnosed in Malatya, Turkey between January 1st, 2001 and December 31st, 2007.

	Number of cases (%)		ρ Value
	Pulmonary (n = 294)	Extrapulmonary (n = 103)	
**Age (years)**			< 0.01
0-15	36 (12.24)	15 (14.56)	
16-30	87 (29.59)	25 (24.27)	
31-45	68 (23.13)	19 (18.45)	
46-60	33 (11.22)	27 (26.21)	
≥ 61	18 (6.12)	11 (10.68)	
Unknown	52 (17.69)	6 (5.83)	
**Gender**			0.11
Male	183 (62.24)	55 (53.40)	
Female	111 (37.76)	48 (46.60)	
**Drug Resistance**			0.37
Any Drug Resistance	88 (29.93)	26 (25.25)	
Susceptible	206 (70.07)	77 (74.76)	
**IS*6110 *Copies**			0.66
≤ 5 copies	75 (25.51)	24 (23.30)	
> 5 copies	219 (74.49)	79 (76.70)	
**Spoligotype Superfamily**			0.20
T	131 (44.56)	53 (51.46)	
Lam	63 (21.43)	14 (13.59)	
H	43 (14.63)	13 (12.62)	
Undefined	35 (11.90)	18 (17.48)	
Other	22 (7.48)	5 (4.55)	
**Cluster**			< 0.01
Clustered	112 (38.10)	20 (19.42)	
Unique	182 (61.90)	83 (80.58)	

### Microbial characteristics and risk factors of EPTB

IS*6110 *RFLP typing showed that one fourth of the study isolates (99/397) were low-copy number isolates having less than 5 copies of IS*6110*. IS*6110 *copy numbers varied from 2 to 15 and 65% of the isolates had 7-12 copies. The distributions of the high copy and low copy isolates were not statistically significantly different between PTB and EPTB groups (Table 1). A combination of the results of both IS*6110 *RFLP analysis and spoligo secondary typing identified 112 (38.10%) of the 294 PTB isolates and 20 (19.42%) of the 103 EPTB isolates as clustered. Unique strains were overrepresented among EPTB cases while clustered strains were overrepresented among the PTB cases (P < 0.01).

Spoligotyping defined a total of nine spoligo superfamilies that include 344 (86.6%) of the 397 study isolates. Fifty three (13.4%) of the spoligotype were not matched in the any spoligotypes in the SpolDB4 database. Ill-defined T (46.3%, 184/397), Latin American and Mediterranean (LAM, 19.4%, 77/397), and Haarlem (H, 14.1%, 56/397) were the most commonly seen superfamilies found in our study sample. Four isolates (1.0%) were determined to be Beijing family strains. There was no statistically significant difference in the distribution of spoligo superfamilies between PTB and EPTB (Table 1). However, it is worth to mention that of the four Beijing strains, one was from a pulmonary case, while three were EPTB isolates.

Eighteen (4.5%) of the 397 isolates were multi-drug resistant (MDR), 96 (24.2%) were non-MDR resistant isolates, and 283 (71.3%) were susceptible to all the drugs tested. Drug resistance was not significantly different between PTB and EPTB (Table 1).

After adjustment for potential confounders in a multivariate logistic regression model, infection of unique strain remained strongly associated with EPTB (Table 2).

**Table 2:** Multivariate logistic regression analysis of 397 TB cases diagnosed in Malatya, Turkey between January 1st, 2001 and December 31st, 2007 to identify demographic and microbial risk factors for having extrapulmonary TB in Malatya

Variable	Adjusted OR (95%CI)*	
Age		
0-15 years	1.00	Referent
16-30 years	0.78	(0.36, 1.71)
31-45 years	0.73	(0.32, 1.65)
46-60 years	1.97	(0.87, 4.50)
≥ 61 years	1.64	(0.60, 4.51)
Unknown	0.22	(0.08, 0.64)
Sex		
Male	1.00	Referent
Female	1.34	(0.82, 2.19)
Any DR	1.00	Referent
Susceptible	1.26	(0.73, 2.18)
IS*6110 *Copies		
≤ 5 copies	1.00	Referent
> 5 copies	0.92	(0.51, 1.67)
Spoligotype superfamilies		
T	1.00	Referent
Lam	0.62	(0.30, 1.27)
H	0.72	(0.34, 1.50)
Undetermined	1.21	(0.59, 2.46)
Other	0.48	(0.17, 1.40)
Clustered/Unique		
Cluster	1.00	Referent
Unique	2.82	(1.59, 5.00)

* Adjusted for all variables

## Discussion

To gain a better understanding of the epidemiology of EPTB in Turkey, we analyzed demographic, clinical and microbial characteristics of 397 TB patients diagnosed in Malatya, Turkey between January 1st, 2001 and December 31st, 2007. About one fourth of the study subjects had EPTB. Different types of EPTB occurred at a varied frequency, with genitourinary and meningeal TB being the most commonly seen types. Age distribution was significantly different (P < 0.01) between PTB and EPTB, people aged 46 and above appeared to have an increased risk for having EPTB. Furthermore, the distribution of different forms of EPTB differed significantly among age groups (P = 0.03), resulting in significantly different age compositions for different specific types of EPTB. *M. tuberculosis *isolates obtained from EPTB cases were significantly more likely to have unique DNA fingerprinting patterns. Different from previous studies of EPTB, in our study, being female was not found to be a risk factor for EPTB. Furthermore, although it was not statistically significant, meningeal and bone and/or joint TB were more commonly observed among the male patients than among the female patients. In contrast, lymphatic, genitourinary, and peritoneal TB cases were predominant among females.

The previous reported proportion of EPTB among all TB cases in different countries varied between 12% and 25.8% [5,9,11,20]. Previous studies conducted in different regions of Turkey showed that the rates of EPTB cases among all TB cases ranged from 3.2% to 53.8% [21-25]. EPTB was found in 25.9% of our study population. Furthermore, the distribution of different forms of EPTB has varied among studies conducted in different populations, including different regions of Turkey. In our study population, genitourinary and meningeal TB was commonly seen EPTB types including 27.2% and 19.4% of the EPTB cases, respectively. In contrast, in several earlier studies conducted in other regions of Turkey, the most frequently seen forms of EPTB were pleural TB [21,22], lymphatic TB [23], and central nerves system TB [26], respectively. A previous study performed on 85 culture-proven EPTB cases in Arkansas showed that bone and/or joint tuberculosis was the most common type of extrapulmonary tuberculosis (27.1%) followed by cervical lymphatic tuberculosis (17.7%) [4]. Another study conducted on 480 EPTB cases (of which 76% were culture positive) in San Francisco showed that lymphatic TB was the most frequent form (45.1%) followed by bone and joint TB (15.6%) and pleural TB (14.3%) [11]. Our study included only culture- confirmed EPTB cases because Turgut Ozal Medical Center mainly accepted patients whose diagnosis require more complicated procedure for TB diagnosis. In contrast, in the Turkish studies mentioned above, the EPTB cases were mainly diagnosed based on clinical and radiological findings. Methodological differences, such as the difference in the inclusion criteria mentioned above, can be one of the possible reasons for the reported variations in the distribution of different forms of EPTB across different studies. However, it was worth to note that in all the EPTB studies conducted in Turkey so far, TB meningitis was found to be one of the most frequently observed type of EPTB.

In previous studies, being female, non-Hispanic black, and HIV infected were found to be the independent risk factors for EPTB [4,11,27,28]. In our study population, HIV screening is not routinely done, therefore we did not have the information regarding the HIV infection status of patients. However, according to the reports of the Turkish Ministry of Health, there were only three confirmed HIV/AIDS cases in Malatya at the end of 2007 [29]. Thus, it is unlikely that the high frequency of EPTB in Malatya was driven by high HIV infection rate in the population. The increasing EPTB up to 42% in 2007 in Malatya [13] may depend on an increasing awareness of EPTB and increasing laboratory facilities in recent years. Additionally as it was indicated previously [4]; the high incidence high dynamics of extrapulmonary tuberculosis may be specific to our study population.

Different from several earlier studies of EPTB [4,11,12], our study did not find that female sex was statistically associated with EPTB. The failure to detect an association between female sex and EPTB in our study could be partly due to the low statistical power associated with the small sample size of the study. In addition, we were unable to identify cases that might have had concurrent pulmonary and extrapulmonary involvement due to lack of information about additional disease sites. This limitation may have also contributed to the failure to identify some previously risk factors of EPTB in our study. Nevertheless, our observation of the differential distribution of a given type of EPTB between men and women suggest the possibility that gender differentials in EPTB exist in the population of Maltya, Turkey.

We found that age distribution was significantly different (P < 0.01) between PTB and EPTB. More than 52% of the PTB patients were in the 16-45 years-age group, while the age distribution among the EPTB cases was bimodal. This observation, together with our observation that the clustering rate was significantly higher among the pulmonary cases than among EPTB (38.1% vs. 19.4% respectively, P < 0.01), suggests that most of PTB cases in this population might be largely related to ongoing TB transmission, given that the associated age group (16-45 years) is known as a high risk group for TB transmission [2]. Different from our study, a previous study conducted in Antananarivo (Madagascar) found no significant differences in the clustering rates between PTB and EPTB groups [30] while another study conducted in San Francisco, the United States showed that young age was an independent risk factor for nonrespiratory TB and only pleural TB among the EPTB was associated with the highest clustering rate [11].

As found in our previous studies using pulmonary isolates [15,31], in the present study, the ill-defined T clade, LAM, and Haarlem were the most commonly found clades among both PTB and EPTB cases. A previous population-based study conducted in Arkansas, U.S.A. found that Beijing family strain infection is statistically significantly associated with EPTB [32]. While the number of the Beijing strains in the current study is too small to be assessed for statistical significance, the majority (3/4, 75%) of Beijing strains identified in our study were related to EPTB is intriguing.

## Conclusions

EPTB accounted for a significant proportion of TB cases in Malatya, Turkey between 2001 and 2007. The current study has provided an insight into the dynamics of EPTB in Malatya, Turkey. However, the risk factors for having EPTB remain to be assessed in future studies using population-based or randomly selected sample.

## List of abbreviations

EPTB: Extrapulmonary tuberculosis; PTB: Pulmonary tuberculosis; TB: Tuberculosis; MDR: Multidrug resistant; RFLP: Restriction fragment length polymorphism; LAM: Latin American and Mediterranean;

## Competing interests

The authors declare that they have no competing interests.

## Authors' contributions

SG collected the data, participated in the design of the study, interpreted the data and drafted manuscript. ZH conceived and designed the study, interpreted the data, and provided critical revision of manuscript for important intellectual content. MA conducted all the statistical analysis, interpreted data, and assisted in the manuscript preparation. MK contributed to the collection of study isolates, patient information and interpretation of the results. ZKA contributed to the isolate collection and clinical classification of study patients. RD contributed to the design of the study, interpretation of the data, drafting and revision of the manuscript.

All authors have reviewed and approved the final version of the manuscript.

## Pre-publication history

The pre-publication history for this paper can be accessed here:

http://www.biomedcentral.com/1471-2458/11/154/prepub
